# Effects of a School-Based Rational Emotive Behavior Therapy (REBT) Intervention on Drive for Thinness and Body Esteem Among Late School-Age Girls

**DOI:** 10.3390/healthcare14070844

**Published:** 2026-03-26

**Authors:** Minji Je

**Affiliations:** Department of Nursing Science, College of Life and Health Sciences, Kyungsung University, Busan 48434, Republic of Korea; francisca325@ks.ac.kr

**Keywords:** body esteem, drive for thinness, late school-age girls, Rational Emotive Behavior Therapy

## Abstract

**Highlights:**

**What are the main findings?**

- A school-based REBT intervention significantly reduced excessive drive for thinness and improved body esteem among late school-age girls.
- Cognitive changes were accompanied by improvements in affect and reductions in disordered eating behaviors.

**What are the implications of the main findings?**

- Targeting drive for thinness and body esteem at an early stage may be effective for school-based prevention.
- REBT can be adapted as a universal preventive framework to support healthy cognitive and emotional development in late school age.

**Abstract:**

**Background/Objectives:** Thinness is widely idealized as a standard of beauty, and late school-age girls are increasingly exposed to sociocultural pressures that may be associated with excessive drive for thinness and maladaptive body esteem. These body-related cognitive distortions often emerge regardless of actual weight status and may precede unhealthy dieting behaviors and emotional difficulties. This study aimed to develop and evaluate a school-based Rational Emotive Behavior Therapy (REBT) intervention designed to modify excessive drive for thinness and maladaptive body esteem among late school-age girls. **Methods:** A quasi-experimental, non-equivalent control group design with repeated measures was employed. Participants were 62 girls in grades 5–6 recruited from two public elementary schools in South Korea (experimental group: n = 30; control group: n = 32). The experimental group participated in a five-week REBT intervention consisting of 10 structured sessions grounded in the A-B-C-D-E model, while the control group received no intervention. Outcomes were assessed at pretest, posttest, and follow-up, including drive for thinness, body esteem, positive affect, negative affect, disordered eating behaviors, and social media overuse. Data were analyzed using repeated measures ANOVA. **Results:** Significant group × time interaction effects were observed for drive for thinness, body esteem, positive affect, negative affect, and disordered eating behaviors, with greater changes observed in the experimental group. No significant group × time interaction was found for social media overuse. **Conclusions:** The school-based REBT intervention was associated with reductions in excessive drive for thinness and improvements in body esteem and was also associated with changes in emotional outcomes and reductions in disordered eating behaviors among late school-age girls. These findings support early cognitive modification as a preventive strategy within school settings.

## 1. Introduction

In modern society, thinness is often idealized as a standard of beauty, and this sociocultural perception exerts a pervasive influence on girls during the period when body image is actively forming [1,2,3]. Late school age, which marks the early phase of adolescence, is a critical stage for the development of body-related cognitions, as girls become increasingly sensitive to peer evaluation and sociocultural norms while experiencing rapid physical and psychological changes [4]. During this stage, girls are repeatedly exposed to images that equate thinness with attractiveness through media, peers, and family members. This exposure may be associated with the development of distorted body-related beliefs, which may be manifested as an excessive drive for thinness and maladaptive body esteem among girls [5,6]. These beliefs are not merely individual concerns but may extend into broader sociocultural phenomena. In recent years, pro-ana online culture has spread among some teenage girls in Korea, glorifying extreme thinness and normalizing unhealthy weight-control practices through social networking services—illustrating how excessive drive for thinness and distorted body esteem can be socially reinforced and shared [2,3]. Importantly, this excessive drive often emerges regardless of actual weight status, suggesting that the desire to be thin is not driven by health-related needs but by a cognitive distortion in which thinness becomes a central criterion for self-worth and social acceptance.

During late school age, many girls begin to desire thinness in the absence of medical necessity, reflecting an excessive drive for thinness that is disconnected from actual health needs. When thinness becomes a salient criterion for self-evaluation, maladaptive body esteem may further reinforce this desire, increasing the likelihood of unnecessary dieting behaviors. Empirical studies have shown that girls with heightened drive for thinness and low body esteem frequently misperceive their weight status and engage in harmful weight-control practices, even when they are underweight or of normal weight [1,3,7,8]. Such practices are associated with adverse physical outcomes, including growth retardation, menstrual irregularities, and metabolic disturbances, as well as psychological difficulties such as depression, anxiety, and suicidal ideation [9,10]. Importantly, the early emergence of excessive drive for thinness and maladaptive body esteem may place children on unhealthy developmental trajectories, increasing vulnerability to later disordered eating and emotional problems, including eating disorders such as anorexia nervosa [8,10].

However, children and adolescents tend to conceal their symptoms, which makes early detection difficult. Treatment requires considerable time and cost, and eating disorders carry a risk of recurrent relapse and chronicity, which may pose a life-threatening danger [9,10]. Therefore, preventive approaches that modify excessive drive for thinness and maladaptive body esteem and promote adaptive emotional and behavioral outcomes among children and adolescents are essential. Drive for thinness and maladaptive body esteem among late school-age girls are not merely perceptual problems but are associated with emotional reactions and behavioral outcomes. These cognitive distortions may lead to disordered eating behaviors such as fasting, binge eating, and vomiting [7], or excessive immersion in comparison-oriented social media environments (social media overuse) [6]. In addition, these cognitive distortions and maladaptive health behaviors have been reported to exacerbate negative affect such as depression, anxiety, and self-blame [11], and to show significant associations with positive affect such as interest and pleasure [12,13]. Thus, body-related irrational beliefs among late school-age girls may form a vicious cycle by mediating complex interactions between emotions and behaviors, suggesting the need for an integrated approach to address them simultaneously.

Rational Emotive Behavior Therapy (REBT) provides a cognitive–behavioral framework for identifying and disputing irrational beliefs and restructuring them into more rational beliefs, thereby facilitating adaptive emotional and behavioral functioning [14,15,16]. From an REBT perspective, excessive drive for thinness and maladaptive body esteem can be understood as belief-level distortions that precede and shape emotional responses and health-related behaviors. Previous studies have demonstrated that REBT interventions are effective in reducing binge eating behaviors and improving body image-related outcomes [17,18,19]. However, these interventions have predominantly been implemented among adolescents or adults and have largely focused on individuals who already exhibit problematic or maladaptive behaviors, such as disordered eating patterns or clinically relevant body image concerns [20,21]. Consequently, prior REBT applications have tended to emphasize symptom reduction or behavioral remediation rather than the early modification of distorted cognitive drives before unhealthy dieting behaviors become established.

Therefore, this study aimed to develop and evaluate a school-based REBT intervention designed to modify excessive drive for thinness and maladaptive body esteem among late school-age girls. In addition to these primary cognitive outcomes, the study examined changes in emotional and behavioral health indicators, including affect, disordered eating behaviors, and social media overuse, as outcomes associated with cognitive modification. By targeting distorted cognitive drives before the initiation of unhealthy dieting behaviors, this study sought to contribute to school-based preventive strategies that support healthy developmental trajectories and reduce the risk of eating-related problems in adolescence.

## 2. Conceptual Framework

**Figure 1:**
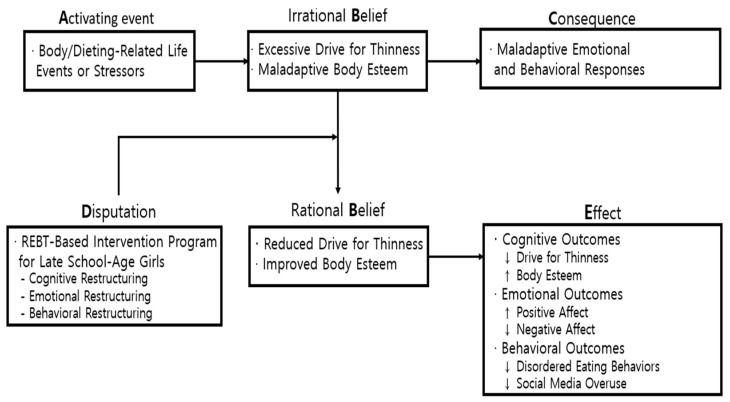
Conceptual framework of the study.

The conceptual framework of this study is based on Rational Emotive Behavior Therapy (REBT), a cognitive–behavioral theory developed by Ellis [15], and is structured according to the A-B-C-D-E model refined by Park [16]. REBT posits that individuals’ cognitions, emotions, and behaviors are deeply interconnected, and that irrational beliefs distort interpretations of life events, leading to maladaptive emotional and behavioral responses. These irrational beliefs, however, can be challenged and modified through systematic disputation, resulting in more adaptive emotional and behavioral functioning. As illustrated in Figure 1, this study applies the A-B-C-D-E model to explain how body- and dieting-related cognitive processes influence late school-age girls’ emotional and behavioral outcomes and how a structured intervention may facilitate change.

In this model, A (Activating Event) refers to body- or dieting-related life events or stressors that elicit maladaptive cognitive responses. These stressors contribute to B (Irrational Beliefs), which in the present study are operationalized as excessive drive for thinness and maladaptive body esteem. Such belief-level distortions give rise to C (Consequences), manifested as maladaptive emotional and behavioral responses, including reduced positive affect, increased negative affect, disordered eating behaviors, and social media overuse.

To address these irrational beliefs, the study implemented D (Disputation) through a structured school-based REBT intervention program designed for late school-age girls. The intervention focused on cognitive, emotional, and behavioral restructuring to help participants identify, dispute, and modify maladaptive beliefs and to regulate associated emotional and behavioral responses.

Accordingly, E (Effect) refers to expected changes following the intervention. These include reductions in drive for thinness and improvements in body esteem as primary cognitive outcomes. They also include emotional and behavioral outcomes, such as increased positive affect, decreased negative affect, and reductions in disordered eating behaviors and social media overuse.

## 3. Methods

### 3.1. Study Design

A quasi-experimental design with a non-equivalent control group and a pretest–posttest structure was employed. Repeated measures analysis of variance (ANOVA) was used to examine changes over time and group-by-time interaction effects. Sample size was calculated using G*Power 3.1.9.7. The parameters were set as follows: effect size f = 0.18 based on a meta-analysis by Jeong & Kim [21], α = 0.05, power (1 − β) = 0.80, three repeated measurements, correlation among repeated measures = 0.50, and two groups. The minimum required sample size was 26 per group (total N = 52). Based on an anticipated 25% dropout rate reported in Cognitive Behavioral Therapy (CBT) studies [22], the target sample size was set at 35 participants per group. During the study, five participants in the experimental group withdrew, and three in the control group did not complete the posttest. As a result, 62 participants—30 in the experimental group and 32 in the control group—completed the study and were included in the final analysis.

### 3.2. Participants

Participants were girls in grades 5–6 enrolled in two public elementary schools located in metropolitan cities in South Korea. Prior to recruitment, approval was obtained from the school principals, and a study announcement was posted on classroom bulletin boards. Students who were interested in participating voluntarily expressed their intention to participate to the researcher or the classroom teacher. Group assignment was determined by school affiliation. The experimental group was recruited from H Elementary School in U City, whereas the control group was recruited from J Elementary School in B City. The two schools were located in different metropolitan cities to minimize contamination between groups and were comparable in terms of student demographics and school characteristics.

Eligibility criteria included being Korean aged 12 to 13 years, enrolled in a regular classroom, and having concerns or experiences related to body shape, appearance, or dieting typical of late school age. Participants were required to be able to communicate in Korean and independently complete self-report questionnaires. Exclusion criteria included previous or current exposure to pro-ana content or eating disorder-related education, therapy, or counseling; a clinical diagnosis of an eating disorder; or cognitive, emotional, or behavioral disorders (e.g., depression, ADHD) that could interfere with participation or data collection. Participants were recruited using a school-based convenience sampling approach. Informed consent was obtained from both students and their parents or legal guardians in accordance with ethical research standards.

### 3.3. Procedure

The study was conducted between December 2022 and March 2023 and followed a three-stage procedure: pretest, intervention, and posttest/follow-up. All assessments were administered in classroom settings at each participating school. Pretest data were collected one week before the intervention. After obtaining informed consent from parents and assent from students, participants completed self-report questionnaires assessing general characteristics and six outcome variables: drive for thinness, body esteem, positive affect, negative affect, disordered eating behaviors, and social media overuse. Questionnaire administration took approximately 20–30 min. The school-based REBT intervention was delivered to the experimental group over five weeks. Posttest and follow-up assessments were conducted five and nine weeks after the pretest, respectively, using the same procedures and instruments. Only participants who completed all three assessments (pretest, posttest, and follow-up) were included in the final analysis; therefore, no imputation for missing data was performed.

### 3.4. Measures

Drive for thinness. Drive for thinness was assessed using the Drive for Thinness subscale of the Eating Disorder Inventory-2, originally developed by Garner et al. [23], revised by Garner [24], and adapted into Korean by Lee [25]. The subscale comprises 7 items rated on a 6-point Likert scale, with higher scores indicating a stronger drive for thinness. Cronbach’s α was 0.70 in the Korean version [25] and 0.89 in the present study.

Body esteem. Body esteem was assessed using the Body Esteem Scale, developed by Mendelson et al. [26] and adapted into Korean by Lee [27]. The scale includes 23 items rated on a 4-point Likert scale, with higher scores reflecting more positive body esteem. Cronbach’s α was 0.92 in the Korean version [27] and 0.81 in the present study.

Positive affect. Positive affect was measured using the Positive Affect subscale of the Positive and Negative Affect Schedule (PANAS), developed by Watson et al. [28] and adapted into Korean by Lee [29]. This subscale comprises 10 items rated on a 5-point Likert scale, with higher scores indicating higher levels of positive affect. Cronbach’s α was 0.88 in the original version, 0.84 in the Korean version [29], and 0.80 in the present study.

Negative affect. Negative affect was measured using the PANAS [28] and adapted into Korean by Lee [29]. It consists of 10 items rated on a 5-point Likert scale, with higher scores reflecting more intense negative emotional experiences. Cronbach’s α was 0.85 in the original version, 0.87 in the Korean version [29], and 0.85 in the present study.

Disordered eating behaviors. Disordered eating behaviors were measured using the Restrained Eating subscale of the Dutch Eating Behavior Questionnaire (DEBQ), developed by Van Strien et al. [30] and translated into Korean by Kim et al. [31]. The subscale comprises 10 items rated on a 5-point Likert scale, with higher scores indicating more disordered eating behaviors. Cronbach’s α was 0.95 in the original version [30], 0.90 in the Korean version [31], and 0.93 in the present study. Although the DEBQ Restrained Eating subscale was originally validated for female adults, previous studies have demonstrated satisfactory internal consistency for DEBQ factors among girls of similar ages. Halvarsson and Sjödén [32] reported acceptable internal consistency of the DEBQ among girls aged 9–10 years, with a Cronbach’s α of 0.77 for the restrained eating subscale. In addition, Van Strien and Oosterveld [33] reported acceptable reliability of the scale among children aged 7–12 years, with a Cronbach’s α of 0.82.

Social Media Overuse. Social media overuse was assessed using the Korean version of the Social Network Service Addiction Proneness Scale, developed by Jung & Kim [34] and modified by Park [35]. The scale includes 24 items rated on a 4-point Likert scale, with higher scores indicating greater overuse. Cronbach’s α was 0.92 in the original version [34], 0.94 in the modified version [35], and 0.93 in the present study.

### 3.5. Intervention

The intervention was a school-based Rational Emotive Behavior Therapy (REBT) program specifically designed to modify excessive drive for thinness and maladaptive body esteem, which were conceptualized as core body-related irrational beliefs in the present study. Grounded in the A-B-C-D-E model of REBT, the program aimed to promote early cognitive modification before the initiation of unhealthy dieting behaviors and associated emotional and behavioral problems among late school-age girls. Program content was developed based on a comprehensive review of the literature on REBT-based preventive interventions and qualitative interviews with four sixth-grade girls who reported strong interest in body shape and thinness. The preliminary program was reviewed and refined by an expert panel consisting of certified counseling psychologists with expertise in cognitive–behavioral therapy, an educational scholar, an elementary school teacher, and nursing scholars. To enhance developmental appropriateness and clarity, the finalized materials were further reviewed by four sixth-grade girls, and minor revisions were made to simplify language and instructions.

The final program consisted of 10 structured sessions delivered twice a week over five weeks, with each 60 min session addressing belief–emotion–behavior processes through which body-related irrational beliefs, including excessive drive for thinness and maladaptive body esteem, operate. Sessions were intentionally sequenced to align with the causal logic of the REBT A-B-C-D-E model, which posits that beliefs—rather than activating events themselves—drive emotional and behavioral responses. Accordingly, the program was structured to first establish awareness and identification skills before introducing disputation techniques. In the early sessions, participants were guided to recognize body- and dieting-related activating events (A) commonly encountered in daily school, peer, and media contexts. They were also guided to identify the associated irrational beliefs (B), operationalized in this study as excessive drive for thinness and maladaptive body esteem. These sessions emphasized distinguishing beliefs from events and making implicit appearance-related assumptions explicit. Subsequent sessions focused on helping participants understand how these belief-level distortions generate maladaptive emotional and behavioral responses (C), such as negative affect, disordered eating behaviors, and social media overuse, thereby increasing perceived relevance and motivation for change. Disputation skills (D) were introduced only after this cognitive foundation was established, enabling participants to apply disputation techniques to personally relevant beliefs rather than engaging with REBT concepts abstractly. Disputation was practiced through cognitive, emotional, and behavioral restructuring strategies, including guided exercises, role-play scenarios, group discussions, and reflective writing. Participants were encouraged to generate rational alternative beliefs, rehearse adaptive responses, and apply these skills to situations that typically triggered appearance-related concerns. Homework assignments were incorporated in selected sessions to reinforce skill application in daily life. The later sessions were designed to consolidate these skills through repeated practice and reflection, with the expected effects (E) reflected in reductions in excessive drive for thinness and improvements in body esteem as primary cognitive outcomes. Changes in emotional and behavioral outcomes—including increased positive affect, decreased negative affect, and reductions in disordered eating behaviors and social media overuse—were conceptualized as associated effects of cognitive modification. An overview of session themes and activities is presented in Table 1.

Participants were divided into five small groups of six to seven students to promote peer interaction and experiential learning. A mobile messaging platform was used to support ongoing group and individual communication throughout the intervention period. All sessions were conducted by the researcher to ensure consistency of intervention delivery. Two trained research assistants, both elementary school teachers, attended each session to support student engagement and maintain a safe and structured learning environment. Prior to implementation, the assistants completed three preparatory training sessions. The sessions took place in a well-equipped classroom at H Elementary School. The control group did not receive the intervention during the study period but was provided with the program workbook after study completion.

### 3.6. Data Analysis

All data were analyzed using IBM SPSS Statistics version 25.0 (IBM Corp., Armonk, NY, USA). Descriptive statistics—including frequencies, percentages, means, and standard deviations—were used to summarize participants’ general characteristics and outcome variables for both the experimental and control groups. The assumptions underlying the statistical analyses were assessed prior to hypothesis testing. Normality of the data was evaluated using the Kolmogorov–Smirnov test. To verify baseline homogeneity between the two groups, chi-square tests or Fisher’s exact tests were applied for categorical variables, and independent *t*-tests were conducted for continuous variables. To examine interaction effects between time and group, repeated measures ANOVA was performed. Mauchly’s test of sphericity was used to assess the sphericity assumption; when this assumption was violated, the Greenhouse–Geisser correction was applied. Internal consistency of the measurement instruments was evaluated using Cronbach’s alpha. Missing data were handled using a listwise deletion approach, and only participants who completed all three assessments (pretest, posttest, and follow-up) were included in the final analysis. Consequently, no statistical imputation for missing data was performed.

### 3.7. Ethical Considerations

The study protocol was reviewed and approved by the Institutional Review Board of the Catholic University of Pusan (Approval No. CUPIRB-2022-053). Written informed consent was obtained from both the participating children and their legal guardians. The researcher provided detailed information about the study’s purpose, procedures, potential risks and benefits, confidentiality, and voluntary participation. Participants were informed that they could withdraw at any time without any disadvantage. All data were anonymized using identification codes. Physical documents were stored in a locked cabinet, and digital files were saved on encrypted, password-protected computers. Survey materials from participants who withdrew were immediately destroyed. Upon study completion, small gifts were given to all participants, and the same educational workbook used in the intervention was provided to the control group.

## 4. Results

### 4.1. Baseline Characteristics

Baseline equivalence between the experimental group (n = 30) and the control group (n = 32) was assessed using chi-square and independent *t*-tests. No significant differences were found in grade, age, height, weight, or BMI category (Table 2).

### 4.2. Baseline Homogeneity Test of Outcome Variables

Independent *t*-tests were conducted to compare pre-intervention scores for the six outcome variables between the two groups. No statistically significant differences were observed in drive for thinness, body esteem, positive affect, negative affect, disordered eating behaviors, or social media overuse (Table 3).

### 4.3. Changes in Outcome Variables Across Time and Groups

Repeated measures ANOVA was conducted to examine group × time interaction effects for the six dependent variables. Significant group × time interaction effects were found for drive for thinness, body esteem, positive affect, negative affect, and disordered eating behaviors. In contrast, no significant group × time interaction effect was found for social media overuse (Table 4).

## 5. Discussion

This study applied the cognitive, emotional, and behavioral disputation techniques of the A-B-C-D-E model of REBT [15,16] to late school-age girls, aiming to modify excessive drive for thinness and maladaptive body esteem, conceptualized as core body-related irrational beliefs. The findings revealed significant group × time interaction effects in cognitive factors, including drive for thinness and body esteem, suggesting that the REBT-based program may have positively influenced irrational beliefs among late school-age girls. These results are consistent with the findings of Pratt & Woolfenden [17], who reported reductions in adolescents’ body idealization and dissatisfaction, and with the systematic review by Guest et al. [19], which examined programs aimed at improving body image among children and adolescents.

The program incorporated cognitive disputation techniques such as belief card activities and sentence restructuring [15,16], which enabled participants to directly examine beliefs underlying excessive drive for thinness and maladaptive body esteem. Sustaining cognitive change requires acquiring conversational and behavioral skills applicable to real-life situations [16,36]. Accordingly, role-play activities were applied to allow participants to experience real-life situations in which appearance-related beliefs could operate and to repeatedly practice rational conversations and behaviors. Late school-age girls tend to show egocentric thinking and strong needs for social acceptance [4]. Given this, the program emphasized self-awareness and restructuring of rational beliefs, which may have been associated with sustained changes in body-related cognitive distortions.

Significant group × time interaction effects were also found in emotional factors, including both positive and negative affect, indicating that changes in excessive drive for thinness and maladaptive body esteem were accompanied by improvements in emotional regulation. In particular, the reduction in negative affect is consistent with prior findings. Yang & Han [18] showed that an REBT-based binge eating intervention alleviated negative emotions among female college students. Similarly, King et al. [20] reported that REBT interventions significantly reduced negative affect, including depression and anxiety.

The program included not only cognitive disputation strategies but also emotional techniques such as emotion card activities, relaxation exercises, and the Letting-go box, enabling participants to recognize emotions and understand their links to underlying beliefs. Through these integrated strategies, participants were encouraged to explore emotional experiences from a belief-oriented perspective rather than simply expressing or releasing emotions. This process may have contributed to reductions in negative affect. In addition, the use of practical emotion regulation strategies applicable in daily life may have supported the maintenance of emotional change.

Moreover, positive affect significantly increased following the intervention. Enhancing positive affect may function as a protective factor in the prevention of eating-related problems [12]. To foster this, the program incorporated activities designed to strengthen positive affect, such as creating a Soothing box, with the aim of building emotional resources rather than solely reducing risk factors. This enhancement of positive affect may have buffered negative emotional responses arising from distorted body-related beliefs and supported healthier emotional regulation. Future research should further examine positive affect enhancement as a preventive intervention strategy.

In behavioral outcomes, significant group × time interaction effects were observed in disordered eating behaviors, whereas no statistically significant changes were found in social media overuse. The reduction in disordered eating behaviors aligns with previous findings indicating that cognitive modification can lead to behavioral change [18,37]. In the present study, cognitive and emotional disputation strategies were implemented prior to providing information about dieting or weight management, thereby facilitating voluntary behavioral change. Through this preventive approach, participants were encouraged to reassess behavioral standards shaped by external expectations, which may have supported reductions in disordered eating behaviors.

However, no significant changes were found in social media overuse. This finding may be partly attributable to contextual factors surrounding the intervention period, including limited external regulation of social media use during the winter vacation. In addition, the use of a group-based messaging platform for program coordination may have reduced the sensitivity of the social media overuse measure to short-term change. These results may also reflect adolescents’ developmental tendency to view social media as an important means of social interaction used for information seeking and peer communication rather than a discrete voluntary behavior [6,38]. Furthermore, as noted by Stice et al. [37], cognitive changes may not immediately translate into measurable behavioral shifts in habitual patterns, such as digital usage, without higher intervention intensity or longer follow-up periods. This is consistent with Gordon et al. [39], who found that a school-based social media intervention produced no significant behavioral outcomes in adolescents, suggesting that deeply habituated patterns of social media use may limit the effectiveness of brief interventions. Future studies should consider more nuanced assessments of social media use that capture qualitative patterns of engagement rather than overall frequency. Moreover, given that social media environments may reinforce excessive drive for thinness and maladaptive body esteem among adolescent girls [2,6], future preventive programs may benefit from incorporating educational and behavioral strategies specifically targeting social media-related cognitive processes.

This study has theoretical and practical significance as a school-based preventive REBT intervention that targeted excessive drive for thinness and maladaptive body esteem during late school age, a critical developmental period for the formation of body-related cognitions. The observed reductions in drive for thinness and disordered eating behaviors in the experimental group suggest that modifying irrational beliefs related to body image may be an effective strategy for early prevention in school settings. Furthermore, the improvements in body esteem and emotional outcomes indicate that interventions simultaneously targeting cognition, emotion, and behavior may support healthier psychological development among late school-age girls, reflecting the core principles of the REBT A-B-C-D-E model and its potential applicability to universal preventive programs for children.

Nevertheless, this study has several limitations. First, it was conducted with a limited sample of elementary school girls in a specific region, which may introduce selection bias and limit the generalizability of the findings. In addition, the quasi-experimental design without random assignment may introduce allocation bias, further limiting the generalizability of the findings. However, baseline equivalence between the experimental and control groups was statistically confirmed to partially reduce potential group differences. Furthermore, the restrained eating subscale of the DEBQ was originally developed for adults. Although previous studies have reported acceptable reliability of the scale among children of similar ages, caution may be warranted when interpreting disordered eating behaviors in younger populations. Second, as all measurement tools were self-report questionnaires, responses may have been influenced by subjective judgment, which may introduce self-report bias. To evaluate changes in cognition, emotion, and behavior more reliably, complementary methods such as parent or teacher observations and digital media use records should be considered. Third, program effects were measured only immediately after the intervention and four weeks later, making it difficult to fully assess long-term sustainability. Future studies should adopt designs including mid- to long-term follow-up. Fourth, although the data were collected between December 2022 and March 2023, the issues addressed in this study, such as excessive drive for thinness and maladaptive body esteem among adolescent girls, continue to be important concerns in adolescent health.

## 6. Conclusions

This study developed and tested a school-based REBT intervention program specifically targeting excessive drive for thinness and maladaptive body esteem among late school-age girls. The experimental group showed significant changes in drive for thinness, body esteem, negative affect, positive affect, and disordered eating behaviors, whereas no statistically significant changes were observed for social media overuse. These findings suggest that early modification of cognitive drives related to thinness and body esteem may contribute to broader emotional and behavioral changes during late school age. This study contributes theoretically and practically by providing empirical evidence for a structured preventive intervention that targets belief-level distortions before the emergence of unhealthy dieting behaviors in school settings. Future research should examine the long-term sustainability of these preventive effects and extend the application of the program to diverse populations and contexts.

## Figures and Tables

**Table 1:** Structure of the School-based REBT Intervention Program.

Session	REBT Component & Theme	Content	Method
1	Program Introduction, Motivation	Program overview and participation expectations Introduction to REBT and the A-B-C-D-E model Icebreaking and personal introductions Participation and confidentiality pledge Homework: Reflective writing on appearance-related beliefs and dieting	Lecture, writing, Sharing
2	A-B-C: Identifying Events, Beliefs, and Consequences	Share reflective writing with peers Apply the A-B-C model to a sample case Analyze one's own experience using the A-B-C model Learn about irrational vs. rational beliefs Evaluate and discuss personal beliefs for rationality	Lecture, case analysis, reflection, discussion, sharing
3	B: Exploring Irrational Beliefs and Preparing for Cognitive Restructuring	Sort irrational and rational beliefs using belief cards Transform irrational beliefs into rational alternatives using sentence restructuring Read reframed rational beliefs aloud Homework: Write dialogue lines for a role-play activity	Lecture, card activity, restructuring, reading
4	D: Cognitive Restructuring	Share role-play homework lines Perform role-plays based on appearance-related beliefs Share reflections from the role-play Compare the strengths and weaknesses of irrational and rational beliefs	Role-play, Expression, discussion, evaluation, sharing
5	B-C: Exploring Emotions Triggered by Irrational Beliefs	Explore a range of emotions using emotion cards Select emotions related to emotions triggered by appearance-related beliefs using emotion cards Express emotions through facial expressions and gestures Discuss reasons behind selected emotional responses Homework: search for an item that improves mood	Lecture, card activity, play, sharing, discussion
6	D: Emotional Restructuring	Share mood-enhancing items brought from homework Learn and practice emotional relaxation techniques Release negative emotions by discarding written thoughts into the Letting-go box Create a personalized Soothing Box with a meaningful item	Lecture, show-and-tell, relaxation, symbolic activity, sharing
7	B-C: Exploring Behaviors Triggered by Irrational Beliefs	Evaluate dieting beliefs and practices and classify them as helpful or harmful Reflect on and assess one's own past dieting behaviors Visualize personal social media usage through graphing activities Homework: Track and graph daily social media usage	Lecture, analysis, graphing, self-monitoring
8	D: Behavioral Restructuring	Discuss personal patterns and motives for social media use Learn appropriate and balanced ways to use social media Discuss balanced approaches to eating and physical activity Review key content through an O/X quiz game	Lecture, quiz, discussion,
9	E: Identifying Effects of Belief Change	Create a personal crown and attach automatic thoughts using thought bubbles Write a three-syllable encouraging phrase to the crown Take a photo wearing the crown and decorate it Homework: List personal strengths excluding appearance	Craft, writing, sharing, photo
10	E: Consolidation and Closure	Share personal strengths written for homework with peers Identify and share positive changes experienced during the program Share encouraging messages with peers using a rolling paper Make a written pledge to apply program learnings moving forward	Reflection, feedback, writing, Sharing

**Table 2:** Baseline Characteristics and Homogeneity Tests between the Experimental and Control Groups (N = 62).

Characteristics	Category	Experimental (n = 30)	Control (n = 32)	χ^2^ or t	*p*
		n (%) or M ± SD	n (%) or M ± SD		
Grade	5th grade	5 (16.7)	9 (28.1)	1.16	0.281
	6th grade	25 (83.3)	23 (71.9)		
Age (years)		12.83 ± 0.38	12.72 ± 0.46	1.08	0.286
Height (cm)		156.06 ± 6.56	153.72 ± 7.85	1.26	0.214
Weight (kg)		48.27 ± 9.29	47.59 ± 7.37	0.32	0.752
BMI ^a^	Underweight	13 (43.3)	7 (21.9)	3.52	0.172
	Normal weight	14 (46.7)	22 (68.8)		
	Overweight	3 (10.0)	3 (9.3)		

Note. Values are presented as n (%) or mean ± standard deviation. M = Mean; SD = Standard Deviation; BMI = Body Mass Index; ^a^ BMI categories were based on age- and sex-specific percentiles for Korean girls aged 11–13: underweight (<5th percentile), normal weight (5th–84th), overweight (≥85th). χ^2^ tests were used for grade and BMI.

**Table 3:** Baseline Comparison of Outcome Variables between the Experimental and Control Groups (N = 62).

Variable	Range	Experimental (n = 30)	Control (n = 32)	t	*p*
		M ± SD	M ± SD		
Drive for Thinness	1–6	3.84 ± 1.12	3.60 ± 1.01	0.87	0.388
Body Esteem	1–4	2.15 ± 0.41	2.35 ± 0.46	−1.81	0.075
Positive Affect	1–5	2.73 ± 0.78	2.81 ± 0.66	−0.42	0.679
Negative Affect	1–5	2.55 ± 0.91	2.27 ± 0.67	1.36	0.179
Disordered Eating Behaviors	1–5	3.28 ± 0.89	3.08 ± 1.06	0.81	0.422
Social Media Overuse	1–4	2.09 ± 0.66	2.11 ± 0.55	−0.14	0.888

Note. Values are presented as mean ± standard deviation. M = Mean; SD = Standard Deviation. Independent *t*-tests were used to compare baseline differences between the experimental and control groups.

**Table 4:** Changes in Outcome Variables over Time in the Experimental and Control Groups (N = 62).

Outcome Variable	Time	Experimental (n = 30)	Control (n = 32)	Source	F	*p*
		M ± SD	M ± SD			
Drive for Thinness	Pre	3.84 ± 1.12	3.60 ± 1.01	Group (G)	20.37	<0.001
	Post	1.84 ± 0.64	3.17 ± 1.10	Time (T) ^a^	47.06	<0.001
	Follow-up	1.69 ± 0.67	3.09 ± 1.28	G × T ^a^	18.50	<0.001
Body Esteem	Pre	2.15 ± 0.41	2.35 ± 0.46	Group (G)	18.76	<0.001
	Post	3.13 ± 0.58	2.41 ± 0.49	Time (T) ^a^	43.30	<0.001
	Follow-up	3.21 ± 0.57	2.49 ± 0.41	G × T ^a^	29.11	<0.001
Positive Affect	Pre	2.73 ± 0.77	2.81 ± 0.65	Group (G)	3.39	0.071
	Post	3.33 ± 0.88	2.81 ± 0.69	Time (T)	8.28	<0.001
	Follow-up	3.29 ± 0.69	2.88 ± 0.72	G × T ^a^	6.30	0.003
Negative Affect	Pre	2.54 ± 0.91	2.27 ± 0.67	Group (G)	2.61	0.111
	Post	2.08 ± 0.76	2.56 ± 1.01	Time (T)	0.34	0.675
	Follow-up	2.06 ± 0.70	2.69 ± 0.90	G × T ^a^	10.30	<0.001
Disordered Eating Behaviors	Pre	3.28 ± 0.89	3.08 ± 1.06	Group (G)	0.83	0.367
	Post	2.65 ± 1.03	3.11 ± 0.91	Time (T)	6.26	0.010
	Follow-up	2.63 ± 0.96	2.94 ± 1.03	G × T ^a^	4.54	0.029
Social Media Overuse	Pre	2.09 ± 0.66	2.11 ± 0.55	Group (G)	0.59	0.447
	Post	1.93 ± 0.60	2.02 ± 0.63	Time (T)	1.59	0.213
	Follow-up	1.96 ± 0.61	2.15 ± 0.57	G × T ^a^	0.67	0.418

Note. Values are presented as mean ± standard deviation. M = Mean; SD = Standard Deviation. Repeated measures ANOVA was used to test group (G), time (T), and group × time (G × T) effects. ^a^ Greenhouse–Geisser correction was applied. *p* < 0.05 was considered statistically significant.

## Data Availability

The data are not publicly available due to ethical restrictions but may be obtained from the corresponding author upon reasonable request.

## Abbreviations

The following abbreviations are used in this manuscript:

REBT	Rational Emotive Behavior Therapy
ANOVA	Analysis of Variance
PANAS	Positive and Negative Affect Schedule
